# Establishing the extent of malaria transmission and challenges facing pre-elimination in the Republic of Djibouti

**DOI:** 10.1186/1471-2334-11-121

**Published:** 2011-05-11

**Authors:** Abdisalan M Noor, Maoulid B Mohamed, Cleopatra K Mugyenyi, Mouna A Osman, Hawa H Guessod, Caroline W Kabaria, Ifrah A Ahmed, Mary Nyonda, Jackie Cook, Christopher J Drakeley, Margaret J Mackinnon, Robert W Snow

**Affiliations:** 1 Malaria Public Health & Epidemiology Group, Centre for Geographic Medicine Research - Coast, Kenya Medical Research Institute/Wellcome Trust Research Programme, P.O. Box 43640, 00100 GPO, Nairobi, Kenya; 2 Centre for Tropical Medicine, Nuffield Department of Clinical Medicine, University of Oxford, CCVTM, Oxford OX3 7LJ, UK; 3 Direction de l'Epidémiologie et de l'Information Sanitaire, Ministère de la Sante, P.O. Box 438, Djibouti, République de Djibouti; 4 Programme National de Lutte Contre le Paludisme, Ministère de la Sante, P.O. Box 1157, Djibouti, République de Djibouti; 5 Centre for Geographic Medicine Research - Coast, Kenya Medical Research Institute/Wellcome Trust Research Programme, P.O. Box 230, Kilifi, Kenya; 6 Department of Immunity and Infection, Faculty of Infectious and Tropical Diseases, London School of Hygiene and Tropical Medicine, Keppel Street, London, WC1E 7HT, UK

## Abstract

**Background:**

Countries aiming for malaria elimination require a detailed understanding of the current intensity of malaria transmission within their national borders. National household sample surveys are now being used to define infection prevalence but these are less efficient in areas of exceptionally low endemicity. Here we present the results of a national malaria indicator survey in the Republic of Djibouti, the first in sub-Saharan Africa to combine parasitological and serological markers of malaria, to evaluate the extent of transmission in the country and explore the potential for elimination.

**Methods:**

A national cross-sectional household survey was undertaken from December 2008 to January 2009. A finger prick blood sample was taken from randomly selected participants of all ages to examine for parasitaemia using rapid diagnostic tests (RDTs) and confirmed using Polymerase Chain Reaction (PCR). Blood spots were also collected on filter paper and subsequently used to evaluate the presence of serological markers (combined AMA-1 and MSP-1_19_) of *Plasmodium falciparum *exposure. Multivariate regression analysis was used to determine the risk factors for *P. falciparum *infection and/or exposure. The Getis-Ord G-statistic was used to assess spatial heterogeneity of combined infections and serological markers.

**Results:**

A total of 7151 individuals were tested using RDTs of which only 42 (0.5%) were positive for *P. falciparum *infections and confirmed by PCR. Filter paper blood spots were collected for 5605 individuals. Of these 4769 showed concordant optical density results and were retained in subsequent analysis. Overall *P. falciparum *sero-prevalence was 9.9% (517/4769) for all ages; 6.9% (46/649) in children under the age of five years; and 14.2% (76/510) in the oldest age group (≥ 50 years). The combined infection and/or antibody prevalence was 10.5% (550/4769) and varied from 8.1% to 14.1% but overall regional differences were not statistically significant (χ^2 ^= 33.98, p = 0.3144). Increasing age (p < 0.001) and decreasing household wealth status (p < 0.001) were significantly associated with increasing combined *P. falciparum *infection and/or antibody prevalence. Significant *P. falciparum *hot spots were observed in Dikhil region.

**Conclusion:**

Malaria transmission in the Republic of Djibouti is very low across all regions with evidence of micro-epidemiological heterogeneity and limited recent transmission. It would seem that the Republic of Djibouti has a biologically feasible set of pre-conditions for elimination, however, the operational feasibility and the potential risks to elimination posed by *P. vivax *and human population movement across the sub-region remain to be properly established.

## Background

In Africa, some countries in the North have achieved malaria elimination in the last 30 years (Libya in 1973, Tunisia in 1979, and Morocco in 2010) and the majority of the region is largely regarded as malaria free despite a few disputed residual foci in Algeria and Egypt. Elimination, however, poses a major challenge in the majority of countries in Africa owing to the intrinsically high transmission intensity within each country's national borders [1] or in the cases of low transmission areas, threats posed by neighbouring high transmission countries [2], operational constraints posed by fragile health systems that may not reach remote foci of infection [2], the sophistication of current health information systems to identify all cases of clinical and asymptomatic malaria [3], and the ability to meet the immediate increases in financing needs that would be diverted from other health problems [4,5].

A recent systematic analysis of available national data has defined possible biological threats (including current and historical receptivity of transmission and connectivity to other countries) and operational threats (including political stability, heath system capacity and access to populations) to elimination across 99 malaria endemic countries [2]. Within Africa the most technically and operationally feasible countries, as judged by a combined ranking, were Botswana, Swaziland, São Tomé and Príncipe, Eritrea, and Djibouti. The Republic of Djibouti was considered the African country with the most favorable combinations of biological risk and operational requirements to reach elimination and its ranking globally was equivalent or better than many countries in the Americas. However, several recent publications have highlighted that despite the fact that 32 countries have declared an elimination ambition [6] few have defined the true biological feasibility of elimination [7] and fewer have defined a combined biological and operational feasibility assessment [6,8]. There have been limited opportunities for countries to assess their technical and operational strengths and weaknesses for elimination unless undertaken as part of very detailed reviews of large amounts of empirical data as implemented recently in Zanzibar, United Republic of Tanzania [9].

National Malaria Indicator Surveys (MIS) have been established largely to provide milestone measurements for reaching endemic control ambitions in high to moderate transmission countries, with parasite prevalence as the main transmission indicator. In low transmission areas this indicator is not efficient [10,11]. Here we present the results of a recent adaptation of a standard MIS to include a national serological survey in the Republic of Djibouti to define the extent of transmission and explore the possibilities of malaria elimination.

## Methods

### National context

**Figure 1:**
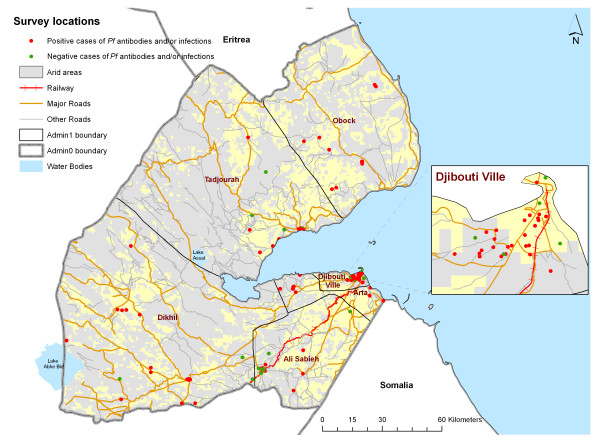
**Map of the Republic of Djibouti showing inland water, roads, railway, administrative regions and major settlements geo-coded in Google Earth and obtained from DEPHA **[64]**, showing location of clusters with persons indentified as sero-positive for *P. falciparum *antibodies and/or RDT-PCR confirmed parasite positive (red) and clusters where no person identified as sero-positive or parasite positive (green) overlaid on 1 × 1 kilometre resolution surface of aridity as defined by GlobCover **[33].

The Republic of Djibouti is a small country of 23,200 Km^2 ^located at the southern reaches of the Red Sea on the Horn of Africa, bordered by Somaliland region of Somalia in the South, Ethiopia in the West and South and Eritrea in the North. It was the last French territory on mainland Africa to gain independence in 1977 with important strategic economic and political value as a port in the Gulf of Aden. The significance of the port at Djibouti for the Ethiopian economy was recognized at the end of the 19^th ^Century when the Emperor declared Djibouti an official outlet of Ethiopian commerce that prompted the development of the Addis Abba-Dire Dawa-Djibouti Railway (Figure 1). This rail link continues to be an important physical connection to Ethiopia.

The majority of the population are represented by either the Issas (ethnically Somalis and connected to clans across the sub-region from Somaliland, the Ogaden, and Yemen) or the Afars (ethnically connected to people of Ethiopia). Other smaller groups exist including people of Arab origin and refugees who migrated into the country during the 1980's and 1990's from the war-ravaged neighboring countries. The population is spatially over-dispersed with 60% of the entire country residing in Djibouti Ville and its suburbs. Five secondary urban settlements exist outside the main city at Arta, Obock, Tadjourah, Ali Sabieh and Dikhil (Figure 1) and are inhabited by 10% of the population; the remaining 30% of the population live in villages or nomadic pastoralists scattered across 90% of the country's semi-arid, volcanic land surface in the hinterland. The last census was undertaken in 2009 and it was estimated that there were 818,159 people [12]. The country is divided into six administrative regions.

There are few rivers but one tributary of the Awash River feeds the inland Lake Abbé on the border with Ethiopia and seasonal *Wadis *feed the extremely saline Lake Assal which is located west of the inlet water of Ghoubet Kharab, and is the lowest point of Africa (Figure 1). Rainfall is sparse and erratic with average annual precipitation of 130 mm with the short rains in March to May and the long rains in July to September. The average temperature in the country is 30°C with the hot season from May to September during which the north eastern monsoon blows. Average humidity is above 60% throughout the year around Djibouti Ville and much less inland [13].

### Malaria history and priorities 2006-2010

The limited annual rainfall and extreme aridity provides a natural barrier to stable, endemic transmission of malaria across the majority of the Republic of Djibouti. At the turn of the last century only two foci of malaria transmission were regarded as significant, Ambouli and Ganaam, on the outskirts of Djibouti Ville [14]. Between 1910 and the early 1970's almost no locally acquired cases of malaria were reported in the Republic of Djibouti [15,16]. From 1988 epidemics of malaria began to appear among refugees from neighboring countries and led to onward transmission among local resident communities [14,15,17]. A study among 144 febrile individuals in 1989 in Ambouli area showed no *P. falciparum *infections but exposure of about 6% to antibodies against the merozoite stage of the parasite [18]. *Anopheles arabiensis *is now accepted as the dominant vector of *P. falciparum *including areas around Djibouti Ville as it expands to the *wadis*, agricultural areas and watering holes around the Ambouli region. Some have argued that *An. arabiensis *was introduced to the area via the rail link to Ethiopia [19]. Two short malaria peaks predominantly occur during the months of May-June and November-December [14].

The Programme National de Lutte Contre le Paludisme (PNLP) was established in 2006 and a National Malaria Strategy was launched the same year which recognized a possible "re-elimination" end game. The stated ambition was to "*interrupt the transmission of malaria and eliminate the disease and its consequences to the socioeconomic development of the country as a major public health problem in Djibouti*" [16]. Despite an anticipated over-dispersion of malaria risks, the aim by 2010 was "*to ensure universal coverage of the profitable interventions of prevention"*. These were to be achieved by increasing the proportion of homes owning 2 insecticide treated nets (ITN) to >= 90% or 60% of children below the age of 5 years protected by an ITN by 2010; introduction of larvivorous fish and Abate for larval control; and urban fogging with insecticides; and case management with first line recommended therapy for uncomplicated malaria as artesunate and sulphadoxine-pyrimethamine combination (AS+SP) combined with increased diagnostic capacities in all health facilities. In addition monitoring systems of health and inter-country and cross-border collaboration were to be established [16]. These strategies were intended to reduce local transmission by half in the whole territory by 2010 with a view to return to zero the number of actively detected cases of malaria and to prevent new transmission from existent cases (stated as a pre-elimination objective). The Republic of Djibouti was successfully awarded by the Global Fund approximately US $ 4 million during round 6 in 2007 and approved a further award during round 9 in 2009 amounting to US $6.6 million to mount pre-elimination efforts up to 2014 [20,21].

### The Djibouti 2008-2009 national MIS

#### Sampling

A population probability sample was used to estimate a sample of households and clusters for each of the six administrative regions reflecting the differences in urban and rural settlement within each region. The estimated required sample was 4,000 households in 160 clusters representing an estimated 24,000 individuals. The sampling indicator used to power the survey was the proportion of children under the age of five years sleeping under ITN based on the expected change from the Multiple Indicator Cluster Survey findings in 2006 [22], predicted household size and age composition, between cluster design-effect of 1.5, 10% non-participation rate and a precision in the estimate of +/- 8.5% derived from a balance between optimized precision and costs [23]. The survey sample was drawn from a sampling frame of 450 urban and rural clusters (defined as census enumeration areas with a minimum of 150 households) provided by the Direction de la Statistique et des Etudes Demographique (DISED) [24]. The sample was then re-adjusted to over-sample regions outside of Djibouti Ville by reducing the sampled clusters for Djibouti Ville to 40% of the overall sample rather than the expected probability sample of 60% and redistribute the 20% proportionately across the other less densely populated regions of the interior. For each cluster, a household sampling interval was selected from the sampling frame such that if a cluster had a total of 75 households, every third household was surveyed. The sampling interval was updated from the village elders during the day of survey and any necessary revisions were made. During the survey each cluster was positioned using a hand-held Global Positioning System (Etrex H, Garmin Inc., USA).

#### Survey procedures

A community sensitization exercise was undertaken a week before the survey via print, radio and television media and local women's groups encouraging support and participation during the survey. Training of all survey coordinators, supervisors and investigators in survey tools and procedures occurred during a two week period in November 2008. The survey was undertaken over three weeks between December 2008 and January 2009 by eight trained teams comprised of a team supervisor, interviewer, nurse and laboratory technician. Three of the survey teams were assigned to Djibouti Ville region and one team for each of the other regions. The survey instruments used during the household investigations were adapted from standard MIS tools developed by the Roll Back Malaria Monitoring and Evaluation Reference Group [24]. Important variations on the standard MIS included ensuring information was captured on all age-groups on ITN use and peripheral blood sampling for malaria parasite and antibody detection in a sub-sample of household members of all ages.

At consenting households a *de facto *census was undertaken, including information on age, sex and use of nets/treated nets and their source. Each household head was also interviewed about assets ownership and education. A random sample of three household members were selected from the household census list using a table of random numbers and consenting individuals were asked to provide a finger prick blood sample. The finger prick sample was used to prepare a rapid diagnostic test (Hexagon *Pf *Malaria Rapid HRPII Test (Human GmbH Inc., Germany) or ParaHit *Pf *Rapid Test (ICT Diagnostics, India) to provide immediate treatment using nationally recommended AS+SP for all positive individuals; a thick and thin blood smear, air dried, stained using 5% Giemsa; and three 5 mm diameter drops of blood on Whatman 3 MM filter paper labelled, prepared and stored at 4°C in dry conditions using procedures described elsewhere [25]. This household sub-sample was also interviewed about travel histories in the last six months, duration and destination and the presence of fever in the last 14 days and on the day of survey.

#### Laboratory methods

##### Serological measures

Serologic markers can be particularly useful in areas of low endemicity, where it may be easier to detect relatively long-lasting antibody responses than a low prevalence of malaria infections in the human population or infected mosquitoes [26-28]. Discs of 3.5 mm diameter which contained approximately 3 μl of blood were punched from each filter paper blood spot and reconstituted in low binding 0.5 ml Corning^® ^Costar^® ^96-well flat bottomed plates (Sigma-Aldrich, St. Louis, MO, USA) [25]. A 1:100 dilution of whole blood (equivalent to a 1:200 dilution of serum) was made by adding 300 μl of reconstitution buffer (phosphate buffered saline [PBS], 0.05% Tween-20, 0.1% sodium azide, pH 7.2) to each well. The plate was sealed, rocked at room temperature overnight, and stored at 4°C until required for the Enzyme Linked Immunoabsorbent Assays (ELISA).

Antibodies were detected simultaneously to *P. falciparum *merozoite surface protein 1-19 (MSP1_19_) and apical membrane antigen 1 (AMA-1) by ELISA using procedures described elsewhere [25]. Antigens were obtained as full-length recombinant proteins (AMA-1) or GST fusion proteins (MSP1_19_). Immulon 4 96-well plates (Dynatech Laboratories, Chantilly, VA, USA) were coated with 50 μl of a combination of *P. falciparum *AMA-1(3D7) and *P. falciparum *MSP1_19 _(K1/Wellcome) at concentrations of 0.5 μg/ml in each well and incubated overnight at 4°C. Wells were washed 3 times using wash buffer (PBS-Tween 20, 0.05%). 150 μl/well of blocking buffer (PBS-Tween 20, 1% skimmed milk powder) was added to each well and the plates were incubated at room temperature for 3 hours. Following three washes with wash buffer, 50 μl/well of a 1:1000 dilution of sera in sample buffer (PBS-Tween 20, 1% skimmed milk powder) was added to each well in duplicate and incubated overnight at 4°C. Positive controls (hyper-immune serum pool) were added in duplicate to each plate in a 4-fold serial dilution from 1:50 to 1:51200. Two negative controls (European malaria-negative volunteer serum) were added to each plate in duplicate at the same concentration as the test serum. The plates were washed 6 times using wash buffer and incubated with 50 μl/well of horseradish peroxidase-conjugated polyclonal rabbit anti-human IgG (Dako, Roskilde, Denmark) diluted at 1:5000 in sample buffer for 3 hours at room temperature. After a further 6 washes, the plates were developed for 15-20 minutes in the dark by the addition of 50 μl/well of SIGMA*FAST*™ *o*-phenylenediamine dihydrochloride (OPD) (Sigma-Aldrich, St. Louis, MO, USA) substrate solution. The reaction was stopped by adding 25 μl/well 2 M H_2_SO_4 _and the absorbance measured at 492 nm.

##### Parasitology

Slides taken during the survey proved to be of inadequate quality on reading at the National Public Health Laboratory and at the WHO's Regional Centre for Excellence in Malaria Microscopy in Oman [Mauolid, unpublished observations]. Therefore confirmation of RDT positive and 50 randomly selected negative cases was undertaken using Polymerase Chain Reaction (PCR) protocols for *P. falciparum*.

A 1-3 mm^2 ^spot from the filter paper was excised using a fresh scalpel blade per sample and put into a 1.5 ml pre-labeled tube at room temperature. The QIAamp^® ^DNA Mini Kit (QIAGEN Inc, Valencia, CA) was used to extract DNA according to the manufacturer's instructions. For the RDT positive samples a second extraction was performed using the ABI6500 semi-automated extraction system. Five positive control samples were produced by spotting a parasite blood culture at 40% haematocrit and 1% parasitaemia. Five negative controls were made from non-infected blood in a similar approach. Real-time PCR using a *P. falciparum *specific probe for the the small subunit of rRNA (18s) gene was carried out using the forward and reverse primers 5'-TGCCGACTAGGTGTTGGATG-3' and 5' GCCCCAGAACCCAAAGACTT 3' respectively, and a TaqMan Minor Groove Binder (MGB) probe 5'-FAM-CATCTTTCGAGGTGACTT-MGB-TAMRA 3' labeled with 5'FAM (6-carboxyfluorescein) and 3'TAMRA (6-carboxytetramethyl-rhodamine) as the reporter and quencher, respectively. 2 μl of DNA was added to each reaction in a final volume of 20 ml with 1000 nM each of the forward and reverse primers, 250 nM of the TaqMan probe, and 10 μl of TaqMan Universal master mix (Applied Biosystems, Life Technologies Corp, Carlsbad, CA). The PCR cycling steps were as follows: 50°C for 2 minutes, 95°C for 10 minutes, and 45 cycles of 95°C for 15 seconds and 60°C for 1 minute. All positive controls had critical threshold (*C_t_*) values below 34 cycles. A cutoff of 38 cycles was used to define positive samples.

#### Analysis

All survey data were entered by four data entry clerks using customized data entry packages in MS Access (Microsoft Corporation, USA) which included consistency checks. A wealth index was then calculated from household assets indicators using principal component analysis [29]. Data were subsequently analyzed using STATA version 11 (Statacorp LP, USA). Sampling weights were applied due to the disproportionate selection of clusters per strata and households within each cluster. An additional weight was developed for the parasitology and serology samples where at least 3 individuals were sampled per household. The overall weights of the samples were equal to product of the inverse of the probability of selection of a cluster, household or individual and cluster adjusted and weighted estimates were computed in STATA using the *svy *command. Visual display of all cluster level attributes was undertaken using the GPS coordinates for each cluster and displayed using ArcGIS 9.3 (ESRI Inc. USA). Inland water bodies were assembled from the Global Lakes and Wetlands Database (GLWD) developed by the World Wildlife Fund [30] and major, minor roads and railway were configured from the National Geospatial-Intelligence Agency [31]. The GlobCover Land Cover product was downloaded from the European Space Agency website [32]. This 300 m resolution land cover dataset was derived from a time-series of Medium Resolution Imaging Spectrometer images acquired from December 2004 to June 2006 [33]. The GlobCover surface was used to define clusters located in extremely arid rural areas that have less than 4% total vegetative cover for more than 10 months in a year and are therefore least likely to support vector larval survival [34,35]. Distances between sampled clusters and aridity, main roads and railway and coastline were computed using the *Spatial Analyst *function in ArcGIS 9.3.

All duplicate Optical Density (OD) values > 0.2 which differed by more than a factor of 1.5 were rejected. To define sero-prevalence duplicate OD values were averaged and converted to titres using the positive control curve on each plate to normalize between plates as described below. The mean positive control ODs were fitted to a titration curve using a sigmoid 3-parameter model in Prism version 5 (GraphPad Software Inc, CA, USA). Antibody titres were estimated by the equation described in [25]. A Gaussian mixtures model was fitted to the sample data to determine the sero-negative and sero-positive populations underlying the distribution of the data [25], each with their own mean and variance. This was done using the MCLUST procedure in the R programming language [36]. Antibody titres were logarithmically transformed to render the component distributions normal. The mean plus 3 standard deviations of the sero-negative group was used as the cut-off for sero-positivity. A single cut-off was generated as antibodies to both AMA-1 and MSP-1_19 _were measured in a single well. The sero-conversion rate (λ) was estimated by fitting a reversible catalytic model to the measured sero-prevalence by age in years using maximum likelihood methods [26,37].

PCR positivity was determined using DNA amplification plots of the samples extracted using both the Qiagen and ABI methods which had good correspondence between methods. The PCR amplification plots were displayed and where curves crossed the critical threshold for parasite densities and their controls was used to determine sample positivity.

A multivariate regression model was fitted using the STATA 11 *svy: logistic *command allowing for clustering and weighting of the data to assess the potential predictors of *P. falciparum *exposure and/or prevalence. The association of this outcome measure with age, residence, use of ITN, fever prevalence within the two weeks of survey, distance to main roads and railway, distance to the coast line and aridity, travel in the last six months and wealth quintile were examined. For each predictor the Odds Ratio (OR), 95% confidence interval (CI), and P-values were recorded. All predictors were adjusted for the effect of the variation between regions. A backwards step-wise approach was used to remove the variable with the highest non-significant P-value at each stage of the regression until a final reduced model was achieved with the variables that showed significant association with *P. falciparum *exposure and/or infection prevalence.

To assess spatial heterogeneity the presence of hot/cold spots of *P. falciparum *antibody/infection prevalence was examined using the Getis-Ord G-statistic [38] and associated Z-scores were computed for each cluster in ArcGIS 9.3 (ESRI Inc. USA) using the *Spatial Statistics *tool. A high/low value of the G-statistic indicates that high/low values of infection and/or exposure are clustered within the study area. To determine the significance of these statistics, Z-scores were used. When the G-statistic is significant, positive/negative Z-scores indicate clusters of hot/cold spots.

#### Ethical approval

Ethical approval for the study was obtained from the Direction de l'Epidémiologie et de l'Information Sanitaire (DEIS) of the Republic of Djibouti. All selected household heads were informed of the purpose of the study and offered opportunities to refuse participation. In addition those providing blood samples were explained the procedures and informed written consent signed for all those agreeing to participate.

## Results

### Defining parasite infection and exposure

**Figure 2:**
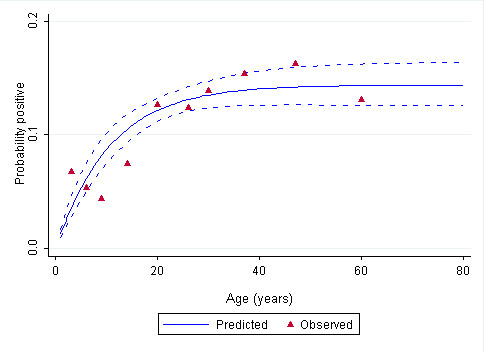
**Age specific sero-prevalence plot of *P. falciparum *antibodies using maximum-likelihood fits from reversible catalytic equilibrium model **[37]. The vertical axis shows the proportion of sero-positive individuals in each age group, the horizontal axis shows the midpoint age. Red triangles represent observed prevalence and blue fitted line is predicted age prevalence curve. λ = 0.0134 (95% CI, 0.010 - 0.018) and ρ = 0.0799 (95% CI, 0.0532 - 0.1200).

Among the 7717 household members randomly selected for parasitological and serological investigation 566 (7.3%) refused participation. A total 7151 consenting individuals in 156 clusters provided blood samples for testing the presence of infection prevalence using RDT with 42 (0.5%) positive for *P. falciparum *infection (Table 1) and confirmed using PCR. The 50 RDT *P. falciparum *negative samples were also confirmed as negative by PCR. Only 5605 (78%) samples from 129 (83%) clusters were available for serological analysis due to interruptions to survey and loss of filter papers. Of the successfully sampled persons, 4769 filter paper samples provided concordant OD data for serological measures of AMA-1 and MSP_19 _*P. falciparum *antigen exposure (Table 1). *P. falciparum *sero-prevalence was 9.9% (n = 517) including 46 children aged less than five years (5 infants aged less than 6 months). Sero-prevalence generally rose with age reaching a peak of 14.2% in the age group 50 or more years with a significant overall trend (χ^2 ^= 68.27, *p*= 0.0001) (Table 1; Figure 2). The seroconversion rate (λ) was estimated by fitting a reversible catalytic model to the measured sero-prevalence by age [26,37] and was estimated to be 0.0134 new exposures per person per year (95% CI: 0.010, 0.018) or about 13 new *P. falciparum *exposures per 1000 persons per year. When the *P. falciparum *sero-positivity and infection prevalence data were combined for the samples that were successfully assayed, overall infection + exposure prevalence was 10.5% with a significant increasing trend with age (χ^2 ^= 67.04, *p*= 0.0001) (Table 1).

**Table 1:** Summary by age of the prevalence of *P. falciparum *antibody exposures and/or infections, period prevalence of fever two-weeks from the survey day and fever on the survey day and use of insecticide treated nets (ITN) the night prior to survey shown as cluster-adjusted and weighted percentages (95%CI) [n/N].

	***P. falciparum *antibody exposure**^**a**^	**Proportion of individuals with *P. falciparum *infection**^**b**^	***P. falciparum *antibody exposure and infection prevalence**^**c**^	Fever prevalence in the two weeks prior to survey	Fever prevalence on the day of survey	Use of ITN
	
Totals	9.9 (7.5 - 12.3) [517/4769]	0.50 (0.28 - 0.73) [42/7151]	10.5 (8.1 - 13.0) [550/4769]	5.2 (4.11 - 6.3) [254/4769]	1.9 (1.4 - 2.4) [91/4769]	14.5 (11.2 - 17.7) [740/4769]
< 5 years	6.9 (3.7 - 10.2) [46/649]	0.23 (0.01 - 0.45) [4/968]	7.1 (3.9 - 10.4) [49/649]	18.5 (12.3 - 24.7) [110/649]	4.3 (2.3 - 6.4) [27/649]	15.8 (10.0 - 21.6) [114/649]
5 - 9 years	4.9 (2.8 - 6.9) [37/703]	0.61 (-0.03 - 1.24) [6/1015]	5.7 (3.4 - 7.9) [43/703]	6.6 (4.5 - 8.6) [46/703]	3.8 (2.3 - 5.2) [26/703]	17.1 (11.8 - 22.5) [126/703]
10 - 14 years	5.0 (2.2 - 7.9) [23/441]	0.34 (-0.06 - 0.74) [3/644]	5.6 (2.7 - 8.5) [26/441]	1.9 (0.6 - 3.3) [9/441]	0.8 (0.0 - 1.6) [5/441]	12.6 (8.6 - 16.6) [55/441]
15 - 34 years	11.4 (8.4 - 14.3) [202/1626]	0.49 (0.16 - 0.82) [14/2454]	11.9 (8.9 - 14.9) [211/1626]	2.5 (1.7 - 3.4) [48/1626]	0.9 (0.4 - 1.4) [16/1626]	12.5 (9.8 - 15.4) [236/1626]
35 - 49 years	14.0 (10.0 - 17.9) [132/833]	0.64 (0.24 - 1.05) [9/1283]	14.7 (10.8 - 18.8) [141/833]	3.4 (1.8 - 5.0) [30/833]	1.6 (0.7 - 2.6) [14/833]	16.7 (12.3 - 21.0) [143/833]
50 + years	14.2 (9.7 - 18.7) [76/510]	0.67 (0.11 - 1.23) [6/773]	15.0 (10.4 - 19.5 [79/510]	2.2 (0.8 - 3.6) [11/510]	0.5 (-0.0 - 1.3) [2/510]	13.6 (9.4 - 17.8) [66/510]
χ^2 ^for trend	68.27, *p*= 0.0001	3.29, *p*= 0.5568	67.04, *p*= 0.0001	268.71, *p*= 0.0001	50.06, *p*= 0.0001	14.23, *p*= 0.0913

For infection prevalence the denominator is all those individuals who gave a finger prick blood sample (n = 7151). For combined infection and antibody prevalence, fever prevalence and ITN use the denominator is the total of samples that had concordant optical density values of *P. falciparum *antibody AMA-1 and MSP-1_19 _antibody exposure (n = 4769).

^a ^Seven individuals had missing age data

^b ^14 individuals had missing age data

^c ^An individual was regarded as a positive case if he/she showed presence of *P. falciparum *infection and/or antibodies in the blood sample. Six of the *P. falciparum *RDT/PCR positive were also positive for antibodies. Three individuals who were RDT positive and PCR confirmed were not successfully assayed. Overall, however, infected individuals showed higher titre responses (mean/median = 196.2/98.8) compared to uninfected individuals (mean/median titres = 161.3/92.2).

### Analysis of risk factors and spatial heterogeneity of parasite infection and/or exposure

**Figure 3:**
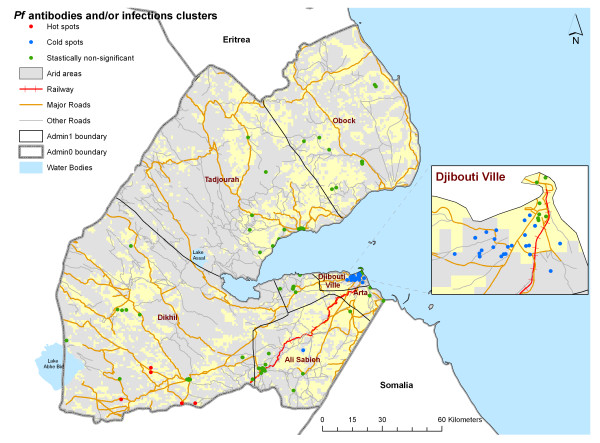
**The location of clusters that are *P. falciparum *antibody/infection prevalence hot spots (red dots, n = 5), cold spots (blue dots, n = 34) and those that don't show significant clustering (green dots, n = 90) relative to neighbouring clusters**. Significance of clustering was analysed using the Getis-Ord G-statistic [38]. Positive Z-scores with significant G-statistic indicated hot spots while negative Z-scores indicated cold spots. The G-statistic was computed using ArcGIS 9.3 *Spatial Statistics *toolbox (ESRI Inc. USA). 33/34 of the cold spots were in Djibouti Ville while all hot spots were in Dikhil region.

The univariate regression analysis showed that there was a statistically significant increase in combined *P. falciparum *sero-positivity and/or infection prevalence if individuals were from the poorest households compared to those from the least poor households (Table 2). No significant differences were observed in prevalence of infection and/or exposure by urban versus rural; ITN use; previous and current fever status; proximity to arid areas, the main roads or railway and the coastline; and travel history in the six months prior to survey within or outside the country (Table 2). The regions of Dikhil (14.1%), Tadjourah (14.0%), Ali Sabieh (11.7%) and Obock (11.1%) had the highest combined *P. falciparum *infection and exposure prevalence, but the overall differences between regions was not significant (Table 2: χ^2 ^= 33.98, *p*= 0.3144). The multivariate regression model showed only age of individual and wealth of household were significantly associated with the combined *P. falciparum *infection and sero-prevalence (Table 3). An increase in age group was associated with about 30% increase in *P. falciparum *exposure and/or infection prevalence (OR = 1.3; 95% CI = 1.2 - 1.6; p < 0.001) (Table 3). An increase in the household wealth quintile was associated with a 20% decrease in likelihood of an individual being positive for *P. falciparum *antibodies or infections (OR = 0.8; 95% CI = 0.7 - 0.9; p < 0.001) (Table 3). The Getis-Ord G-statistic for spatial clustering of *P. falciparum *antibody/infection prevalence was significant for 5 clusters with positive Z-scores (hot spots) and 34 clusters with negative Z-scores (cold spots) while 90 clusters returned values that suggest non-significant clustering (Figure 3). All the clusters of high prevalence were located in Dikhil region while 33/34 clusters of low prevalence were in Djibouti Ville Region. The analysis of the presence of cold/hot spots of parasite prevalence and sero-prevalence separately showed no significant cold/hot spots of the former while the pattern for the latter was similar to that of combined parasite prevalence and sero-prevalence (data not shown).

**Table 2:** Predictors of combined *P. falciparum *antibody and infection prevalence shown as cluster-adjusted and weighted percentages (95%CI) [n/N]

	*P. falciparum *antibody exposure and infection prevalence	χ^2 ^, p-value
**Rural vs Urban**	12.8 (7.7 - 18.0) [188/1291] vs 9.9 (7.1 - 12.6) [362/3478]	7.89, *p*= 0.2906
**Region **		
Ali Sabieh	11.7 (4.1 - 19.3) [57/454]	
Arta	10.5 (6.2 - 14.9) [25/222]	
Dikhil	14.1 (7.0 - 21.3) [109/711]	
Djibouti Ville	8.1 (4.8 - 11.4) [172/2032]	
Obock	11.3 (5.6 - 17.1) [68/595]	
Tadjourah	14.0 (6.0 - 21.9) [119/755]	33.98 *p*= 0.3144
		
**ITN use vs not used**	12.2 (6.5 - 17.8) [100/740] vs 10.30 (7.9 - 12.6 [450/4029]	2.31, *p*= 0.4456
		
**Fever in last 14 days Yes vs No**	9.0 (4.1 - 14.0) [26/254] vs 10.6 (8.2 - 13.1) [524/4515]	0.63, *p*= 0.5207
		
**Fever on the day of survey Yes vs No**	15.5 (6.3 - 24.7) [14/91] vs 10.5 (8.1 - 12.9) [536/4678]	2.35, *p*= 0.1780
		
**Cluster located in arid area Yes vs No**	12.0 (8.4 - 15.7) [343/2727] vs 9.7 (5.8 - 11.6) [207/2042]	13.83, *p*= 0.1607
		
**≤0.1 km vs > 0.1 km to arid area**	11.5 (8.2 - 14.9) [362/2987] vs 9.0 (5.7 - 12.3) [188/1782]	7.44, *p*= 0.3069
		
**≤0.5 km vs >0.5 km to main road/rail**	10.2 (6.7 - 13.7) [274/2456] vs 11.0 (7.6 - 14.3) [276/2313]	0.74, *p*= 0.7549
		
**≤7 km vs > 7 km to the coast line**	9.5 (6.7 - 12.4) [276/2466] vs 12.6 (8.3 - 17.1) [274/2303]	0.01, *p*= 0.9910
		
**Travelled anywhere in the last 6 months yes vs no**	10.5 (7.2 - 13.9) [60/403] vs 10.6 (7.0 - 14.1) [490/4366]	3.03, *p*= 0.3020
		
**Most poor vs Least poor**	15.0 (9.0 - 21.0) [171/1063] vs 6.3 (3.4 - 9.2) [64/973]	41.83, *p*= 0.0039

**Table 3:** Multivariate regression results of the predictors of combined *P. falciparum *antibody exposure and/or infection prevalence shown as cluster-adjusted and weighted Odds Ratio (95%CI) and P-value of the start full model and final reduced model.

	Start Full model		Final reduced model	
	**Odds Ratio (95% CI)**	**P-value**	**Odds Ratio (95% CI)**	**P-value**
**Age category**^**a**^	1.4 (1.2 - 1.6)	0.0001	1.4 (1.2 - 1.6)	0.0001
				
**Urban vs Rural**	0.9 (0.4 - 1.7)	0.660		
				
**ITN use vs not used**	1.0 (0.6 - 1.6)	0.996		
				
**Fever in last 14 days Yes vs No**	0.9 (0.6 - 1.5)	0.792		
				
**Cluster located in arid area Yes vs No**	1.4 (0.8 - 2.4)	0.214		
				
**≤0.5 km vs >0.5 km to main road/rail**^**c**^	0.9 (0.5 - 1.5)	0.631		
				
**≤7 km vs > 7 km to the coast line**^**c**^	0.8 (0.5 - 1.3)	0.406		
				
**Travelled anywhere in the last 6 months yes vs no**^**c**^	1.1 (0.7 - 1.8)	0.361		
				
**Wealth quintile**^**b**^	0.7 (0.6 - 0.8)	0.0001	0.8 (0.7 - 0.9)	0.0001

At each stage of the regression a predictor variable (one with the highest non-significant P-value) was removed from the model in a backwards step-wise approach until all variables in the model had a P-value of less than 0.05.

^a ^The age grouping shown in Table 1 was used during the regression. 14 individuals had missing age data.

^b ^Individuals were classified into the wealth quintile of their resident household.

^c ^Median distances were used

## Discussion

We present here the results from the first national MIS in sub-Saharan Africa to include combined measures of *Pf *parasitological and serological prevalence among all age groups, undertaken in the Republic of Djibouti. *P. falciparum *parasite prevalence was 0.5% and *P. falciparum *sero-prevalence to MSP-1_19 _and/or AMA-1 was 9.9% (Table 2). Sero-prevalence significantly increased with age and the countrywide *P. falciparum *sero-conversion rate (λ) was estimated to be approximately 13 new exposures per 1000 persons per year. Among the 517 individuals who were positive for *P. falciparum *antibodies, 46 were children under the age of five years suggestive of recent malaria exposure. Over 76% of these children were from Tadjourah, Dikhil and Obock regions (data not shown). Overall sero-prevalence in the oldest age group was below 15%. In 2008 among 2896 patients with malaria-like symptoms attending government run clinics with laboratory facilities were tested for presence of infection, 119 (4.1%) were reported positive using microscopy and majority of these cases (74%) were seen in Djibouti Ville [PNLP, unpublished data]. The combined PCR validated *P. falciparum *RDT positivity and sero-prevalence showed some evidence of between region variations with Djibouti Ville showing the lowest rates (8.1%). These regional differences were overall not statistically significant. This study therefore shows that the risks of *P. falciparum *malaria are low across the Republic of Djibouti but majority of clusters surveyed, 102/129 (79%), reported at least one individual who was positive for *P. falciparum *antibodies and/or peripheral blood infection (Figure 3). Although between region variations in exposure was not statistically significant, there appeared to be micro-epidemiological heterogeneity in sero-prevalence with 5 clusters located in Dikhil region showing relatively higher sero-positivity (Figure 3). Residence, aridity, distance to major road/railway and coastline, use of ITNs, recent and present fever histories and travel were not statistically significant predictors of infection and/or sero-prevalence (Table 2). Increasing age and decreasing household wealth were the only two variables significantly associated with increasing *P. falciparum *infection and sero-prevalence independently and in combination.

Sero-epidemiological studies carried out in Tanzania on AMA-1 and MSP-1_19 _showed that the two antigens provided estimates of seroconversion rates that were independently correlated with entomological-based estimates of *P. falciparum *exposure in the same areas [26,39]. AMA-1 has highly immunogenic properties reflecting recent exposure [40,41] while MSP-1_19 _antibodies saturate over longer periods of exposure representing cumulative risks [26]. Combining exposure to both antigens in a single ELISA test is likely to provide a more robust measure of parasite exposure in areas of low parasite prevalence. However interpretation of sero-positivity from optical density values of titres measured through ELISA has an unknown sensitivity and specificity and will depend largely on the approaches taken to classify positivity. Here we elected not to use traditional three standard deviations above established control sera but have used a mixture model method which has previously been used for defining sero-positivity to measles and rubella [42-44]. By using the population itself to define sero-positivity we remove any bias due to population-specific immunoglobin levels, nutritional or co-infection history. However, defining sero-positivity still requires further work to either define their sensitivity, eliminate the need for a cut off or improve approaches to defining it.

From a sampling perspective the largest limitation of the MIS was that it was powered to detect changes in ITN coverage and not parasite exposure. This represents a perennial problem for national MIS across Africa designed to detect increases in coverage measures and not declines in exposure. There is a recommendation however by partners to measure changing parasite prevalence through national surveys every 3-5 years [45]. Using the period prevalence of parasitaemia and/or sero-positivity and the between cluster variability from the survey undertaken in the Republic of Djibouti in 2008-09 we estimate that one would require testing a total population of over 22,000 to achieve a +/- precision of 5% within a point estimate. As prevalence becomes rare the costs of sampling to define parasite exposure therefore increase substantially.

The field investigations of parasite prevalence included two types of HRP-2 RDTs specific only to *P. falciparum *and these, and a random sample of 50 RDT negatives, were validated using PCR resulting in exactly similar results. It is widely held in the Republic of Djibouti that *P. vivax *constitutes less than 5% of all malaria infections [16], however this remains to be investigated properly as vivax infections are often missed during routine clinical microscopy [46]. We have not established *P. vivax *prevalence during the national survey and due to funding constraints we were not able to undertake large-scale PCR-based investigations of vivax and other rare species on the filter paper samples. All neighboring countries connected to the Republic of Djibouti also have *P. vivax *transmission. *P. vivax *is harder to detect, treat and eliminate [47]. The national guidelines on malaria case-management for the Republic of Djibouti are not clear on recommendations for the diagnosis and treatment of vivax malaria [48].

In 2009 the Republic of Djibouti submitted a proposal to the GFATM for five years support to achieve a malaria pre-elimination status by 2015. About 6.6 million USD was approved in June 2009 but the agreement is unlikely to be signed before mid-2011 (personal communication, The Global Fund). Our findings support the biological possibility for elimination reported elsewhere [2] but do not examine the operational feasibility of elimination. Interestingly the GFATM support from round 6 and round 9 continues to promote universal coverage of ITN [20,21]. ITN coverage was 1.4% among children in 2006 [23] and rose in 2008-09 to 19.6% among children aged less than 5 years, with 41.5% of households owning at least one ITN and 14.4% of the entire population using them the night before survey [22]. Increasing ITN coverage to over 80% would continue to be a huge resource outlay for little obvious biological benefit where malaria risk is intrinsically low and spatially over-dispersed. A more logical approach might involve targeted vector control with combinations of larval and adult mosquito target interventions in response to improved vector breeding and/or clinical case detection and cartography [49]. Mathematical models suggest that targeting the minority who contribute the majority to transmission in low transmission settings is likely to yield larger returns [50-52]. Although there was a generally limited clustering of antibody/infection prevalence in Djibouti, there are 4 regions (Tadjourah, Dikhil, Obock and Ali Sabieh) which contribute to the majority of cases. However a migration from blanket coverage to targeted intervention is predicated on a robust and timely surveillance system transitioning from routine clinical treatment with incomplete monthly and quarterly reporting to a model based on daily or weekly aggressive, infectious disease surveillance supported by public awareness campaigns and universal parasitological testing. Malaria is not a notifiable disease in Djibouti and case reporting is embedded within the routine health information system which suffers from perennial under-reporting and data quality problems typical of most health information systems in Africa [53-55]. Surveillance precedents already exist in the Republic of Djibouti for polio and cholera and new infectious disease surveillance initiatives are being developed to track West Nile and Dengue viral infections [Amar Abdo, personal communication]. Malaria surveillance also needs to become an up-regulated disease reporting system with the ability to detect, intervene and eliminate residual foci of malaria infections as and when they emerge. The round 9 proposal includes 0.48 million USD to strengthen surveillance but deciding on how best to develop and implement malaria surveillance systems for elimination has a much weaker evidence-based platform than ITN delivery [3].

Although travel histories in the last six months were not associated with *P. falciparum *infection or sero-prevalence in this study, human population movement is likely to be a significant challenge to elimination in the Republic of Djibouti. Several authors have highlighted the role played by the rail route to Ethiopia in the recent re-emergence of malaria transmission in Djibouti [14,56,57]. Between 700,000 and 1.4 million passengers use this rail route each year [58]. Residents of Djibouti Ville often migrate during the months of May-September to avoid the excessive heat to Hargeisa in Somaliland or Dire-Dawa in Ethiopia. The road from Djibouti Ville to Addis via Galafi has recently been upgraded with funding from the World Bank and the European Union to increase transportation of goods and people between countries [58]. Djiboutian armed and police forces train in Ethiopia and are stationed as part of UN peace keeping in Cote D'Ivoire. Thousands of foreign military personnel are stationed in Djibouti Ville and engage in regional travel. Therefore the significance of human population movement for the risks of imported infectious diseases, including malaria, should not be under-estimated and may be an important contributor to disease hot spots in the country. Although recent reports show significant reductions in malaria infection prevalence in Ethiopia [59], Eritrea [60], Somalia [61] and Yemen [62], the connectivity between the Republic of Djibouti and these countries demonstrates the importance of constantly reviewing the origin and destination risks against regional human population movement to define and contain importation risks [63]. Use of mobile phone data to track population movements internally and externally [63]; screening arriving passengers for malaria at the border entry points and recording of their detailed travel history; and redesigning household survey sampling and questionnaires to capture detailed travel histories of individuals tested for malaria infections are possible approaches to assessing the impact of human population movement on malaria in Djibouti. Work is ongoing to assess the theoretical impact of human population movement on malaria transmission in Djibouti using a combination of travel history data from surveys; air passenger origin and destination data; and estimates of malaria risk of origin and destination [Deepa Pindolia, Personal Communication].

## Conclusion

This study demonstrates the potential of adapting standard national MIS to collect information not only on the prevalence of malaria infections but also serological markers of exposure which are important in determining transmission in areas of low endemicity [26]. The financial cost associated with the materials required to collect dry blood spots for serology and subsequent analysis were relatively small. The additional survey time as a result of collecting the samples on the filter papers appeared minimal. However, it was not possible to conduct a full cost-effectiveness analysis of this adaptation of a standard MIS as this was beyond the scope of this study. The biggest challenge, perhaps, is less to do with actual cost of including a serological component in standard MIS but the expertise required to implement ELISA to investigate exposure. This serological method for malaria is highly specialised and currently implemented largely by research groups. Potentially, however, training of technicians at national public health laboratories in malaria ELISA, as was done in Djibouti for this study, could mitigate this constraint.

The results of the survey support the apparent low case reporting of *P. falciparum *in the Republic of Djibouti and at face value the country may appear to be poised for elimination, a state enjoyed across the country between 1910 and the 1970's [15,16]. *P. falciparum *elimination would be possible if surveillance and response to foci of infections were substantially improved drawing on experience and systems established for polio and other rare, notifiable infectious diseases. Detailed investigations into the extent of *P. vivax *transmission are also needed for a comprehensive understanding of the possibility of elimination [47]. This would require careful planning through a feasibility analysis as undertaken recently in Zanzibar [9] and long-term uninterrupted political support and financing [8]. At present the national malaria control programme has managed to secure some funding to support salaries from the GFATM round 6 funding for a short period but no funds since 2008 to implement any activities [Hawa Guessod, Personal Communication]. The signing and releasing of funds from round 9 has yet to materialize although it is anticipated that a larger national survey will be undertaken in 2012 during which an RDT that can detect both *P. falciparum *and *P. vivax *will be used in addition to collecting blood samples on filter papers for multi-species PCR and ELISA [Hawa Guessod, Personal Communication]. This and the proposed future studies will substantially improve our understanding of malaria transmission in Djibouti, but without a carefully defined plan, staff or funding elimination of malaria in Djibouti remains only a biological possibility. The threshold of parasite prevalence seen as the benchmark for deciding whether to move on to malaria elimination or sustain low endemic control is seen as 1% parasite prevalence [3,11]. But to detect this level of prevalence requires large and expensive survey samples. The threshold for serological markers remains unclear although the absence of exposure among children under the age five years would be a good indicator that a country is approaching pre-elimination [3]. An alternative equivalent index is case incidence of 1 per 1000 persons at risk [3,26]. To reliably estimate this index, there is need for accurate passive and active case detection data over several years [11]. This requires a properly functioning national malaria surveillance system based on a quality assured diagnostic capacity to provide the accurate information on whether Djibouti has achieved the biological threshold for elimination.

## Competing interests

The authors declare that they have no competing interests.

## Authors' contributions

AMN was responsible for study design, data cleaning, analysis, interpretation, drafting and production of the final manuscript. MBM was responsible for the serological analysis of samples in the laboratory and data assembly and contributed to the final manuscript. CKM was responsible for the analysis of the optical density data, interpretation of results and contributed for the final manuscript. MAO, HHG, IAA contributed to the survey design, data assembly and cleaning and contributed to final manuscript. MN was responsible for the polymerase chain reaction analysis of all positive and selected negative samples and contributed to the final manuscript. JC and CJD were responsible for the development of ELISA methods, interpretation of results and contributed to final manuscript. MJM contributed to the statistical analysis of the optical densities, was responsible for the PCR of positive and negative samples and contributed to the final manuscript. RWS was responsible for overall scientific management, analysis, interpretation and preparation of the final manuscript. All authors read and approved the final manuscript.

## Pre-publication history

The pre-publication history for this paper can be accessed here:

http://www.biomedcentral.com/1471-2334/11/121/prepub
